# Multi-Reception Strategy with Improved SNR for Multichannel MR Imaging

**DOI:** 10.1371/journal.pone.0042237

**Published:** 2012-08-03

**Authors:** Bing Wu, Ye Li, Chunsheng Wang, Daniel B. Vigneron, Xiaoliang Zhang

**Affiliations:** 1 Department of Radiology and Biomedical Imaging, University of California San Francisco, San Francisco, California, United States of America; 2 University of California San Francisco/University of California Berkeley Joint Graduate Group in Bioengineering, University of California, Berkeley/San Francisco, California, United States of America; 3 California Institute for Quantitative Biosciences, San Francisco, California, United States of America; Cornell University, United States of America

## Abstract

A multi-reception strategy with extended GRAPPA is proposed in this work to improve MR imaging performance at ultra-high field MR systems with limited receiver channels. In this method, coil elements are separated to two or more groups under appropriate grouping criteria. Those groups are enabled in sequence for imaging first, and then parallel acquisition is performed to compensate for the redundant scan time caused by the multiple receptions. To efficiently reconstruct the data acquired from elements of each group, a specific extended GRAPPA was developed. This approach was evaluated by using a 16-element head array on a 7 Tesla whole-body MRI scanner with 8 receive channels. The *in-vivo* experiments demonstrate that with the same scan time, the 16-element array with twice receptions and acceleration rate of 2 can achieve significant SNR gain in the periphery area of the brain and keep nearly the same SNR in the center area over an eight-element array, which indicates the proposed multi-reception strategy and extended GRAPPA are feasible to improve image quality for MRI systems with limited receive channels. This study also suggests that it is advantageous for a MR system with N receiver channels to utilize a coil array with more than N elements if an appropriate acquisition strategy is applied.

## Introduction

The demand for higher signal-to-noise ratio (SNR) and more accelerated acquisition in magnetic resonance imaging (MRI) and spectroscopic imaging (MRSI) in humans continues to fuel interest in the integrated technique of ultrahigh magnetic field and parallel imaging. In order to achieve higher SNR and acceleration rate in parallel imaging, an RF coil array with a large number of dense-spaced receive elements is required despite the RF design difficulties [1]–[25]. It has been shown that SNR of images has a dependence on the number of receive elements with respect to the object of interests [11], [26], [27]. The increase in system receiver channel count allows the use of coil arrays with a large number of coil elements. Small-diameter surface coils not only benefit for SNR in the locations near the coil, but also benefit for SNR in deep tissues [28], [29]. Furthermore, array with large amount of receive channels allows for increased parallel imaging accelerations and improved spatial resolution compared to nonparallel imaging techniques [11], [30]–[33].

However the benefits of these large arrays are limited to those systems that have enough receiver channels to fully utilize the arrays. Most MR systems today have only eight receive channels or less. In order to use a large or massive array on a receiver limited system to achieve optimized SNR, appropriate signal combination can be done by using specific hardware before the signal reaching the receiver. Correlated method [34] was developed by Spence et al to demonstrate the feasibility. Mode matrix [35] and eigencoil array [36] concepts proposed by Reykowski et al and King et al, respectively, might be another means of using hardware to reduce the number of receivers required and maintain the optimized SNR.

In this work, we propose and investigate a new method utilizing the multi-reception strategy to improve SNR at ultra-high fields. In this method, coil elements are separated to two or more groups under appropriate grouping criteria. To compensate for additional scan time caused by the multiple receptions, parallel imaging is performed group by group with accelerated acquisition. A specific GRAPPA method is developed to reconstruct the data acquired from elements of each group. This approach is experimentally investigated by using a 16-element head array and an 8-element head array on a 7T whole-body MR scanner equipped with eight receive channels. The two coil arrays have the comparable physical size and similar structure. The *in-vivo* experiments demonstrate that with the same scan time, the 16-element array with twice receptions and acceleration rate of 2 can achieve significant SNR gain in the periphery area of the brain and keeps nearly the same SNR in the center area over the 8-element array. The proposed method is dedicated to applications using MR systems with limited receiver hardware. In addition, it also suggests that for a MR system with N receiver channels, it is advantageous and beneficial to utilize a receiver coil array with more than N elements if an appropriate acquisition strategy is applied.

## Materials and Methods

**Figure 1:**
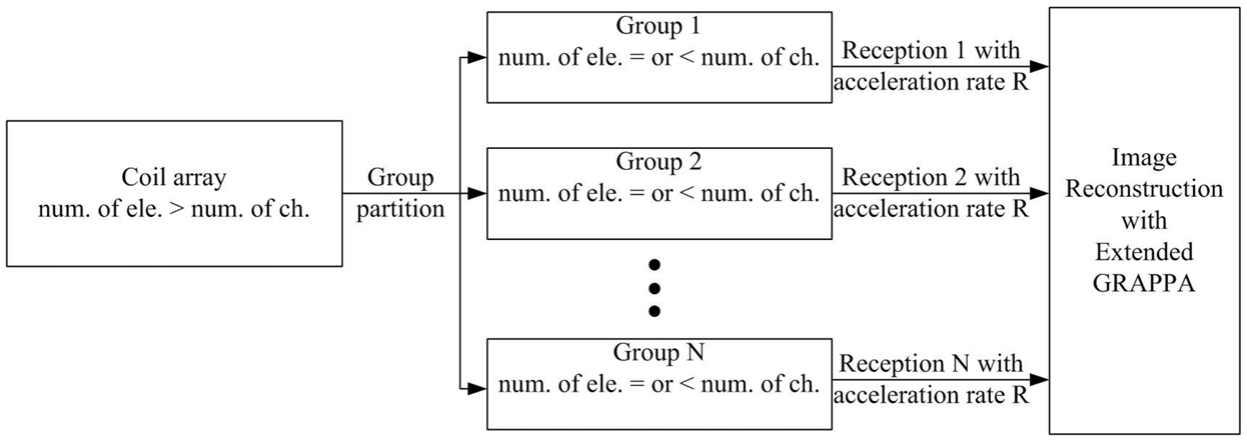
Flow chart of the multi-reception strategy.

Figure 1 schematically depicts how this approach works. Element grouping should be carefully considered for multiple receptions. After coil grouping, the number of coils in each group is equal to or less than the number of receive channels. Each group is switched to the receive channels sequentially during the multiple signal receptions. To speed the imaging, reduced number of phase encoding steps in k-space is applied for each reception, and then parallel MRI technique is used for reconstruction. In this paper, we presented element grouping criteria and proposed an extended GRAPPA for the image reconstruction.

The SNR of parallel imaging at acceleration rate R, *SNR*(*R*), can be calculated as [30]:


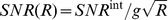
(1)

where *SNR^int^* denotes the intrinsic SNR of fully sampled imaging using sensitivity-weighted optimum phased array combination. The degradation factor 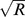 is caused by under-sampling since in Fourier imaging it is obvious that 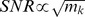 (*m_k_* denotes the number of sample points in k-space). The other noise enhancement factor *g* is usually called geometry factor because it is strongly dependent on the geometry of the coil array, such as the number of coils, the dimension and orientation of each coil element, the distance between coil elements, etc. G-factor also depends on the acceleration rate, the slice orientation and phase encoding direction. This equation was originally developed for SENSE typed parallel imaging. Equivalently, a theoretical description for estimating the noise enhancement in GRAPPA reconstruction has been established [37]. The GRAPPA g-factor can be derived directly from the GRAPPA reconstruction weights.

It is well known that the averaging increases the SNR by the square root of the number of scans averaged. This is because the received NMR signals in *N* receptions have identical amplitudes and phases, after *N* times of averages, signal voltage increases *N*-fold. While noise power also increases *N*-fold, so noise voltage increase 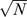 times [38], thus 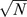 fold SNR increase is achieved.

In the case that the *N* repetitions are received from *N* groups of dense-placed arrays instead of the same phased array, the received signals from each reception, however, may have variable amplitudes and phases, consequently the combined signal voltage is equal to or less than N times of each of signal voltage. SNR of the combined images from N different groups, *SNR*(*N*), decreases with the geometrical deviation of the coil groups. Therefore we have,


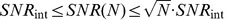
(2)

In the extreme case when the *N* groups are totally overlapped, it equals to one array with *N* NEXs (number of excitations), and SNR can definitely increase 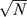
fold. Oppositely if the groups of arrays are separated enough so that no signal enhancement is achieved after the combination of *N* time receptions, the local SNR does not increase at all. Combining equation (1) and (2), we have,


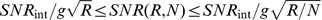
(3)

where *SNR*(*R*,*N*) denotes SNR of combined images from N different groups at reduction rate of R.

**Figure 2:**
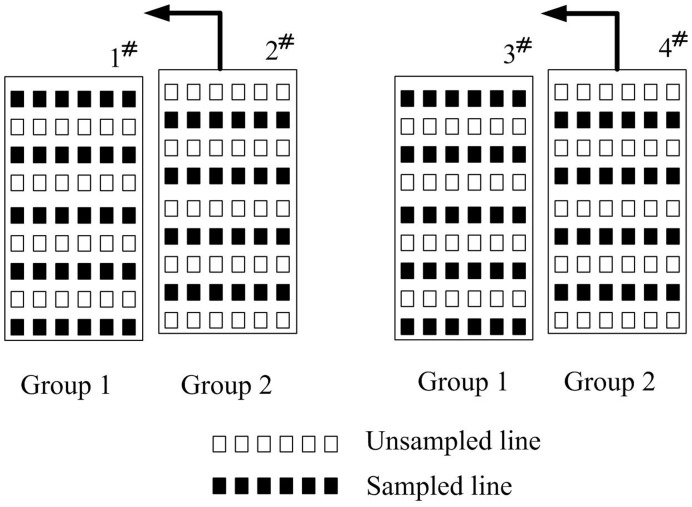
4-element phased array divided into two groups for 2 receive channel system.


Figure 2 shows an example to better understand the equation 3. In the case of a 4-element phased array, the 4 elements are divided into two groups for 2 receive channel system. Elements 1 and 3 belong to group 1, and elements 2 and 4 belong to group 2. Acceleration rate 2 is conducted to reduce the scan time. In group 1, odd numbers of phase encoding lines are sampled while in group 2 even numbers are sampled. If elements 2 and 4 are approaching to elements 1 and 3 respectively, until they are totally overlapped, it is equal to a phased array of two elements, 1 and 3, with fully sampled k-spaces. Thus based on equation 3 we have,

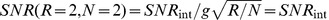
(4)

Equation 4 shows that 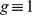
when two groups of coils are totally overlapped. It is an interesting conclusion compared with the conventional parallel imaging conclusion in which the coil overlapping is usually undesired due to the degraded g-factor [26], [33], [39]. If coil elements in different groups are not totally overlapped, GRAPPA method can be applied to fit the missing points.

Further analysis with noise correlation matrix may help understand the SNR improvement by the coil grouping. In an M-channel phased array which is separated into N groups, the noise correlation matrix ψ can be calculated as


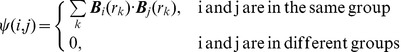
(5)

where i and j are coil element index, k is voxel index, *r_k_* is voxel location and ***B*** is the magnetic field flux density of a coil element. For the coil elements in the same group, the noise correlation keeps the same, while for those in different groups the noise correlation is zero due to multiple receptions. In order to achieve optimal SNR, the coil elements with high noise correlation should be separated to different groups. Generally, adjacent coil elements have higher noise correlation. When the coil elements are overlapped, the noise correlation increases. Therefore, the adjacent coil elements should be separated into different groups to improve SNR. Given that the values of noise correlation matrix were determined mainly by the overlapping of magnetic fields of each coil element in the sample [26], the moderate amounts of inductive coil coupling should not degrade parallel imaging performance of a given coil array [40]. For an optimally decoupled array, appropriate coil grouping can still improve the parallel imaging performance.

### 1. Extended GRAPPA

GRAPPA [32] is currently a leading parallel imaging technique which essentially combines several advantages of the previous improvements to SMASH [31], including auto-calibration, coil-by-coil reconstruction and incorporating multiple data blocks [41]–[45]. In addition, GRAPPA has significantly extended the utilization of acquired k-space signals to derive missing signals in the multiple lines from all channels that are incorporated to produce each missing line in k-space for each single channel. However, the data interpolation scheme is still limited in that the data included to reconstruct each specific missing point is restricted within the phase encoding direction. Such limitation is not intrinsic with the k-space based parallel MRI. In fact, each acquired k-space point can be incorporated in reconstruction, including the data points in frequency encoding direction [46], [47]. Some studies illustrate that using the nearest neighboring points in the PE (phase encoding) as well as FE (frequency encoding) direction can achieve less interpolate fitting error than the regular GRAPPA, especially for volume array, for example human brain array [46],


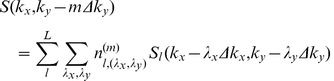
(6)

where S (k_y_) is the signal received by an individual coil corresponding to the k_y_^th^ PE position in the signal space; 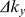
is the PE step; *m* represents the order of harmonics and 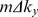
represents a shift by an integer number of steps from the 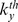
PE positions; *l* and *n* are the number of cols and blocks respectively. *λ_x_* and *λ_y_* are numbers related to the relative shifts from the acquired points to the target point.

**Figure 3:**
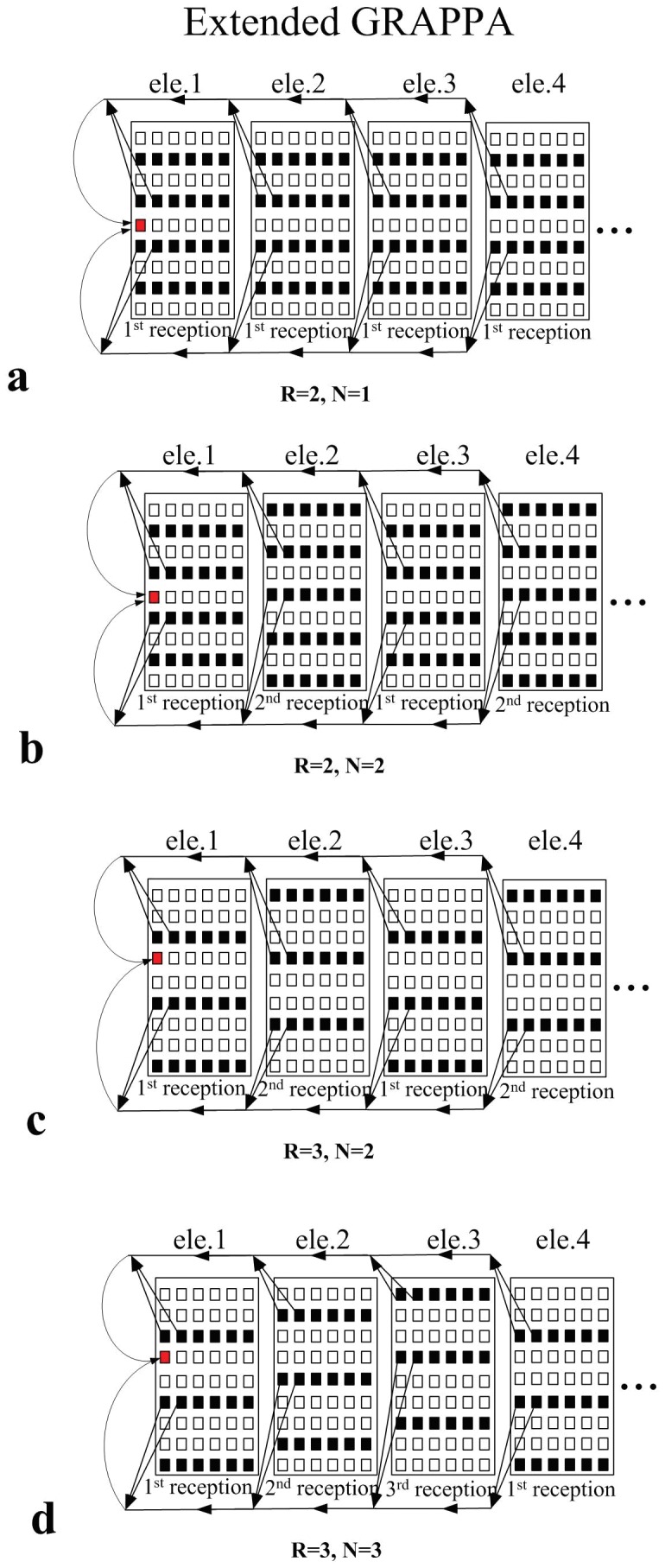
Graphic illustration of the extended GRAPPA.

However, for the phased array with multiple under-sampled receptions (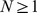
and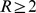), the acquired k-space PE lines for different receptions may be also different. This dedicated k-space sampling scheme together with extended GRAPPA method is illustrated in Figure 3. Figure 3a shows the case of only one reception (N = 1), which is reconstructed using the regular GRAPPA described in [46]. When two receptions (N = 2) are involved and R = 2, to keep the same scan time as the fully sampled scan without average, the acquired PE lines of the first reception and the second reception can be staggered as depicted in Fig. 3b. The extended GRAPPA is applied in which the points in PE and FE direction are incorporated for missing point fitting. Figure 3c shows the fitting method with larger acceleration factor (R = 3) for the N = 2 case and Figure 3d shows the R = 3 case with more receptions (N = 3). The synthesis of the missing lines in the extended GRAPPA can be expressed as:
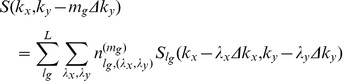
(7)


### 2. Hardware

**Figure 4:**
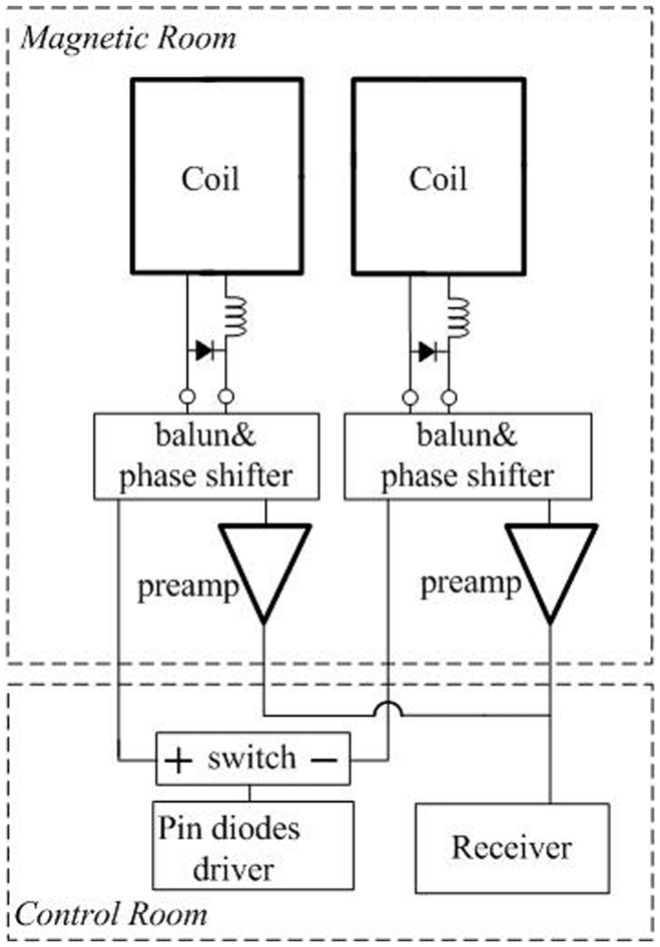
Hardware modification for multiple receptions.

Figure 4 shows one possible solution of hardware modification for this approach. Two coil elements are allocated into one unit, connecting to one receive channel. An electrical switch is inserted between the PIN diode driver and the two coil elements. During transmission phase, both of the coils are forward biased with DC current produced by the driver board. During the first reception, one coil element is inversed biased by changing the DC direction from the switch, so it receives signal normally while the other coil element is still forward biased. During the second reception, the polarity of DC current is changed so that the other coil works. Through this simple circuit, coil elements are grouped and enabled sequentially. To simplify the experiment, we manually switch the connection to receiver to verify the possibility of this approach, which will not impact the imaging quality compared with automatically switching.

### 3. Group Partition for Nova 16-element Brain Array

**Figure 5:**
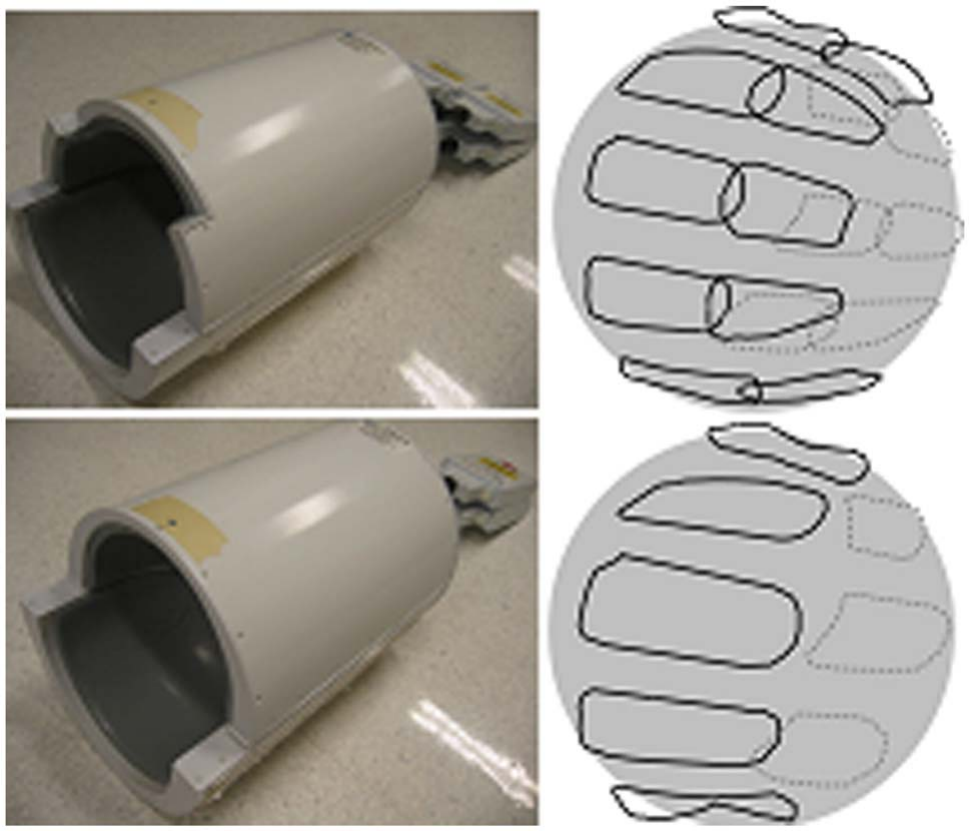
Photos and schematics of Nova 16-element and 8-element human brain receive-only arrays for 7T. Both coil arrays have under-lapped elements in axial plane. 8-element array has one row, while 16-element array has two overlapped rows.

Two human head coil arrays [39] (Nova Medical, Wilmington, MA) with 16-element and 8-element were used to testify the presented method. Those arrays were specifically designed for GE 7T MR system, and with almost the same dimension (shown in Figure 5). The 16-element receive array consisted of sixteen small elements placed on a curved splittable whole brain former. The dimensions of this array were 20 cm long in LR (left-right) direction, 21 cm long in AP (anterior-posterior) and 20 cm long in SI (superior-inferior) direction. Each receive-coil element was approximately 7 cm long by 5 cm wide and tuned to 298 MHz with multiple distributed capacitors. The elements were gapped around axial plane and overlapped along SI direction. Low-input impedance preamplifiers were used for coil decoupling. The 8-element receive array had almost the same dimension with the 16-element array. Each element was approximately 12 cm long by 5 cm wide.

A transmit-only birdcage coil was employed for spin excitation, which was a shielded 16-rung high pass birdcage coil of diameter 30 cm and length 20 cm. The dimensions of the shield were 37.5 cm in diameter and 30 cm in length. The shield was slotted to minimize possible eddy currents and was open at both ends.

**Figure 6:**
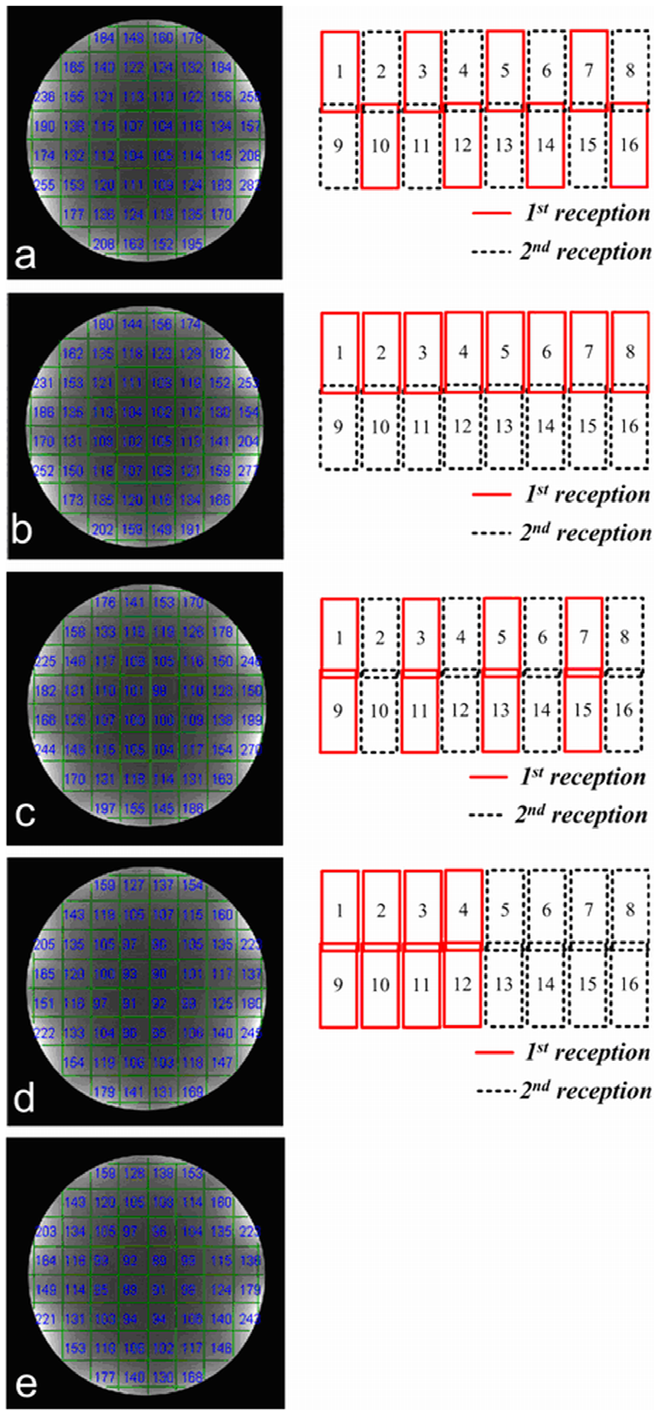
SNRs of silicone oil phantom images of 16-element Nova array with various group partitions: a-d, combined images with 8 receive channels and 2 receptions; e, combined image with single reception and 16 receive channels. It shows that non-overlapped and non-adjacent coils in the same group achieve higher SNR. The image SNR in (a) has 16% SNR gain than (e), while partition in (d) only provides a little SNR benefit.

According to Equation 2, SNR gain was expected for multiple receptions over that of single reception. To quantitatively evaluate the relationship of coil grouping and SNR enhancement, Nova 16-element array with different grouping options were tested in a scanner using its 8 receive channels. The grouping options were demonstrated in Figure 6 (right column). To clearly describe the grouping method, the layout of the 16-elements coil was plotted into a plane in Figure 6.

To quantitatively evaluate the relationship of coil grouping and SNR enhancement, four group partitions of Nova 16-element array were tested using 8 receive channels to compare their SNR gain from two-time receptions. The grouping methods were as follows: in group a, no adjacent or overlapped coils in the same group; in group b, only adjacent (but not overlapped) coils in the same group; in group c, no adjacent but only overlapped coils in the same group; in group d, both adjacent and overlapped coils in the same group. In group e, 16 receive channels with single reception was used as reference. An 18 cm diameter silicone oil (with a low dielectric constant of ∼2.2) sphere phantom was used to eliminate possible dielectric resonance effect in high field MRI. High-resolution phantom experiments were carried out using Spin Echo sequence (5 mm slice thickness, TR/TE  = 1000/3.4 ms, BW = 10.2 kHz) with 512×512 fully sampled matrix. In this work, we used two successive acquisitions for the SNR measurements. Local SNRs in the blocks of 48×48 matrix are calculated, and such local SNRs are determined as the ratio of average signals in the blocks and standard derivation (SD) of the signal in the difference image.


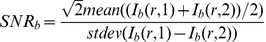
(8)

where 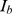 represents signal intensity in each block.

High-resolution human head and noise images (with RF amplifier disabled) were acquired by each grouping option. The signal and noise images were reconstructed with Roemer’s optimal SNR method. Gradient echo (GRE) sequence with 20° flip angle and 512×512 (fully sampled) matrix was used. Additional noise scans were performed by turning off the RF power amplifiers (i.e. no excitation). The noise images reconstructed by using the parallel imaging algorithm were then used to calculate the noise distribution.

Fitting errors of extended GRAPPA and regular GRAPPA described in [46] were also calculated with variable group partitions for 7T human brain images, in which phase encoding direction was towards L-R direction. For GRAPPA-based reconstructions, 10 ACS lines were acquired for R = 3. All of the images were reconstructed offline using reconstruction routines programmed in MATLAB. The scan time for the fully gradient encoded acquisition (R = 1) was about 2 min 12 s. Fitting error images were obtained by subtracting GRAPPA reconstructed images and a fully sampled image with R = 1. Minimum artifact power (AP) was used to evaluate the reconstruction performance [48],


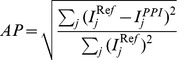
(9)

where 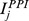 represented the *j^th^* pixel intensity in the accelerated image, and 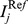 represented the *j^th^* pixel intensity in the corresponding R  = 1 image.

## Results

Four group partitions and the associated SNR maps are shown in Figure 6(a)–(d). SNR of the regular image with 16 receive channels and single reception is also shown in Figure 6(e). The group partition in Figure 6(a) provided the highest SNR gain over other partitions, and approximately 16% SNR enhancement over that from a single received 16-element array with 16 channels. The SNR gain decreases to 13% and 9.7% with the coil grouping in Figure 6(b) and (c), respectively. For the group partition in Figure 6(d), nearly no SNR gain is achieved. The results revealed that the SNR enhancement from multiple receptions was highly dependent on the noise correlation of the coils in the same group. The non-overlapped and non-adjacent coils in the same groups (in Figure 6(a)) were able to achieve higher SNR after combination than that of the overlapped and adjacent element groups.

**Figure 7:**
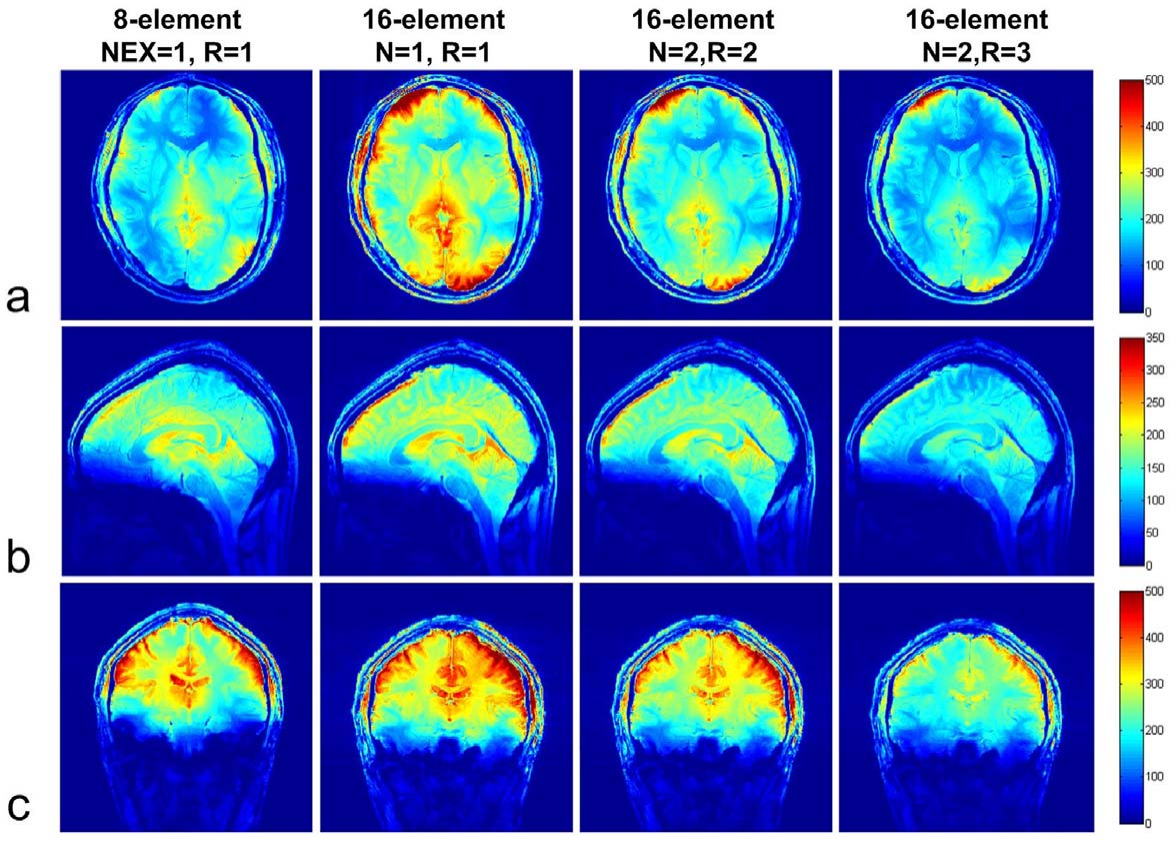
SNR maps derived from eight-element Nova array, sixteen-element Nova array with 16 receivers, sixteen-element Nova array with 8 receivers (N = 2, R = 2) and sixteen-element Nova array with 8 receivers (N = 2, R = 3) at (a) axial plane, (b) sagittal plane and (c) coronal plane. Note that 1.15–1.25 SNR decrease for R = 2 and 1.30–1.50 SNR decrease for R = 3, lower than 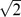
and
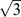
respectively. It is mainly due to the multiple receptions, additional ACS lines acquired for GRAPPA.

The human head SNR maps with axial, coronal and sagittal plane were acquired with the coil grouping shown in Figure 6a (right column). SNR maps from the 16-element array with N = 1 R = 1 (normal scan with 16 receive channels), N = 2 R = 2 (with 8 receive channels), N = 3 R = 2 (only using 6 receive channels), together with the eight element head array N = 1 R = 1 (normal scan with 8 channels) were shown in Figure 7.

Compared to the 8-element array, SNR gain from the 16-element array was as much as 1.7-fold in the periphery of the brain, and typically 15% gain in the deeper region. When the receive channels were reduced to 8, the SNR from 16-element array N = 2, R = 2 is slightly lower (∼4%) over 8-element array in the center of the brain, but still has up to 41% gain at the periphery. After accelerating the 16-element array to 3 and keeping N to 2, it still provided similar SNR as 8-element array at the periphery of the brain, although 46% SNR less at the brain center.

**Figure 8:**
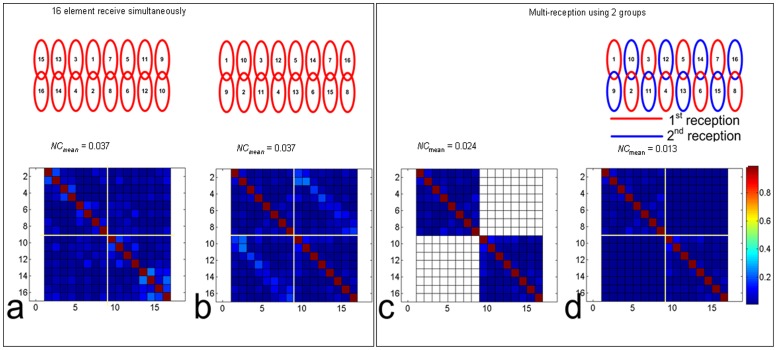
Multiple receiption strategy with proper coil grouping shows the significant noise correcation (NC) improvements, which is helpful to understand the SNR enhancement with this method. The noise correlation matrix (**a**), after re-arrangement with the optimized coil grouping, the largest values are relocated into the I and III quadrants (**b**). After dual receiption, those large values in I and III quadrants are then removed (**c**), and filled with 0 (**d**).

Figure 8 showed the significant noise correcation improvements from the dual reception with the proper grouping. After re-arrangement with the optimized coil grouping, the largest values of the noise correlation were relocated into I and III quadrants, the dual reception then removed those values and replaced with zero. Noise correlation mean value was significantly reduced by 65%.

**Figure 9:**
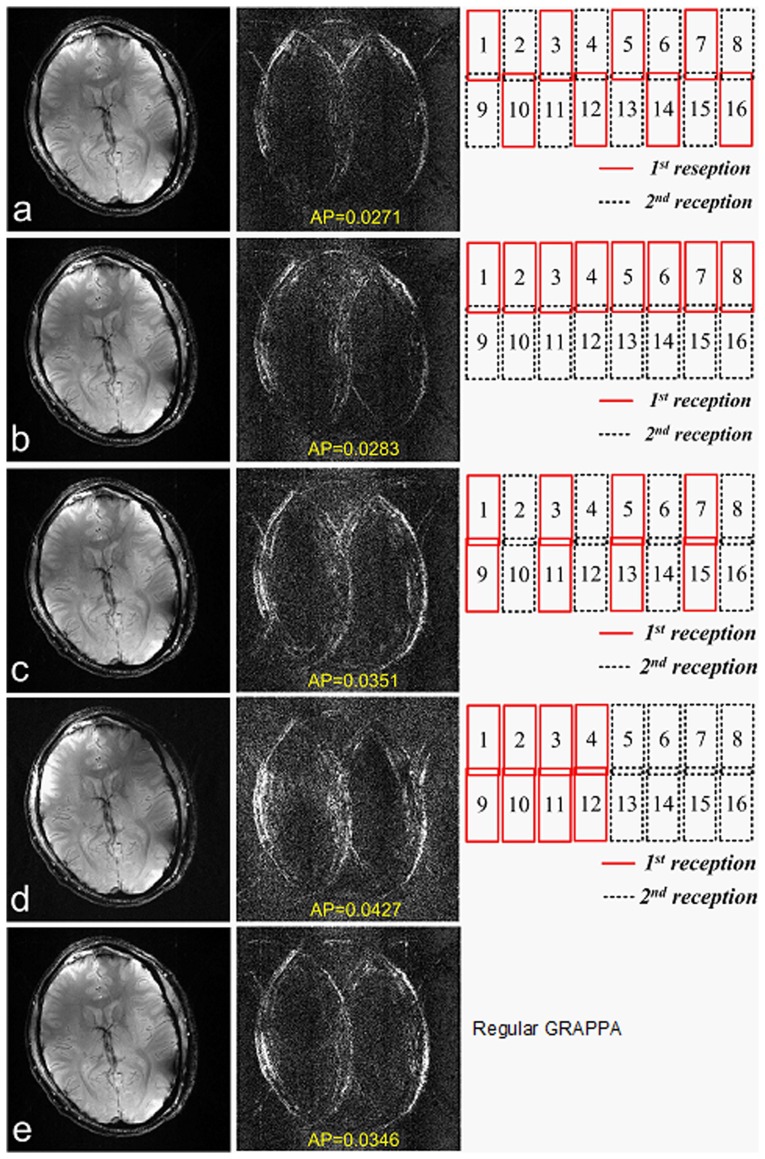
Images, fitting errors and group partitions of 16-element Nova brain array with extended GRAPPA. Accelerate rate R is 3. Group partition (**a**) and (**b**) which avoid overlapped coils in groups, can achieve less fitting error (<20%) than the regular GRAPPA in (**e**). Group partition (**c**) and (**d**) which have overlapped coils in groups, lead to more artifacts in images than that of the regular GRAPPA. 10 ACS lines are chosen, which is acquired inplace and used for the final images. Kernel size is 4, which is composed with two points above and two points below (similar to the method shown in reference 31).

The experiment results revealed that the fitting error of extended GRAPPA was highly related to coil group partitions. Figure 9 (a)–(d) illustrate the reconstructed brain images, error images and the calculated AP values based on extended GRAPPA with R = 3. Figure 9(e) shows the images with regular GRAPPA [46] as a baseline. AP value in Figure 6a was approximately 21.7% lower than that in Figure 6e. It revealed that the artifacts were well controlled. AP in Figure 9(b) was also 18.2% lower than regular GRAPPA. It appeared that the non-overlap coil elements in each groups significantly reduced fitting errors. However, in Figure 9 (c) and (d), the extended GRAPPA led to 1.4% more and 20.0% more artifacts than regular GRAPPA, demonstrating the group partitions with overlapped coil elements in groups decreased the parallel imaging performance.

## Discussion

The multi-reception strategy with extended GRAPPA is proposed to improve image quality in parallel MR imaging in vivo. Human brain images acquired on a 7 Tesla whole-body MR system using a 16-element array with reduction factor R = 2 and number of excitation NEX  = 2 as well as by 8 element array with R = 1 and NEX  = 1 are compared. The experiments show that the 16 element array improves SNR in the peripheral human brain and achieves nearly the same SNR in deep brain region. The proposed method is particularly useful to improve imaging performance for MR imaging where MRI scanner has a limited receive channel number. In addition, this study also suggests that for a MR system with N receiver channels, it is advantageous and beneficial to utilize a receiver coil array with more than N elements to gain SNR and parallel imaging performance [34], [49].

The reconstruction results illustrate that the SNR gain from the multiple reception strategy and the fitting error from extended GRAPPA are highly dependent on the coil element grouping. The maximum SNR gain can be obtained by properly choosing the coil elements for each group set (shown in Figure 6a), which implies that grouping criterion is important to improve the performance of the multi-reception strategy. With the same grouping strategy, under-sampled k-space with extended GRAPPA achieves the minimum reconstruction artifacts.

Since the proposed method requires multiple receptions, the technique might be sensitive to motion or other causes for misregistration between acquisitions. The use of higher acceleration rate can be expected to alleviate the potential misregistration problem. In addition, the proposed multiple reception method increases the complexity of data acquisition, which needs a dedicated k-space sampling scheme. It also needs more complex hardware such as phased array with more elements/channels (than receiver channels of MRI system) to achieve better imaging performance.
